# Aquaculture Navigates Through Troubled Waters

**DOI:** 10.1289/ehp.117-a252

**Published:** 2009-06

**Authors:** David A. Taylor

Industrialized aquaculture (an umbrella term for various methods of domesticated fish production) is the world’s fastest-growing animal food production system and recently surpassed wild catch as the source of the majority of the world’s fish consumption. Around the world, fish consumption averages 16.4 kg per person per year, according to *Fishery and Aquaculture Statistics 2006*, the latest yearbook from the United Nations Food and Agriculture Organization. The glory days of global wild fish catches—which increased from just under 20 million metric tons in 1950 to more than 90 million metric tons in 2005—are over. According to figures from the National Oceanic and Atmospheric Administration (NOAA), wild populations of 46 fish species in U.S. waters were overfished as of late 2008, meaning the capacity of these fisheries to continue producing maximum sustainable yield is in jeopardy.

Critics of the aquaculture industry say it has no place in sustainable food production, pointing to its record of overusing wild fish to feed farmed stock and its effects on surrounding marine systems. Others say many of these criticisms are unfounded or no longer applicable, and that in the face of growing demand for food—for both basic sustenance and the unique health benefits attributed to seafood—aquaculture is a necessary part of any long-term solution. Despite many environmentalists’ misgivings about the industry, the only option for providing more healthy seafare while possibly relieving the pressure on ocean stocks is a better aquaculture, says Mike Rust, who supervises the aquaculture research program for NOAA in Seattle.

## Pressure to Produce

On 21 January 2009 the U.S. Food and Drug Administration (FDA) released for a 90-day public comment period a draft report on the risks and benefits of commercial fish consumption. According to the draft report, a review of various studies led the authors to conclude that fish oil consumption slightly decreased the risk of major coronary events and had significant secondary prevention benefits. For instance, in one 2004 study fish intake once a week was associated with a 13% decrease in stroke risk. Omega-3 polyunsaturated fatty acids appeared to be responsible for the benefits, and the authors of the draft report wrote that these benefits outweighed the considerable risks of neurodevelopmental damage to children caused by prenatal exposure to methylmercury in fish.

But a 17 March 2009 article in the *Canadian Medical Association Journal* by David Jenkins and colleagues from the University of Toronto took another view of the science. These authors concluded that weaknesses in study design rendered even the strongest evidence for the benefits of increased fish oil consumption—including findings from a 1999 study suggesting a 15% benefit in the prevention of cardiovascular disease that could be reduced even further with the incorporation of other healthful lifestyle changes—far from conclusive. The authors questioned, moreover, whether these inconclusive findings were worth the damage that increased fish consumption would wreak on wild fisheries. Moreover, they wrote that aquaculture was “unlikely to resolve the problem,” citing environmental damage caused by fish farms and the risk of exposure to elevated levels of polychlorinated biphenyls (PCBs) and dioxins in farm-raised fish.

These arguments also lie at the heart of the debate on a proposed organic standard for fish products. In November 2008 a working group of the National Organic Standards Board (NOSB) made recommendations to the U.S. Department of Agriculture (USDA) on criteria for adopting an organic standard for wild and farmed fish. Besides codifying “best practices,” an organic standard represents an effort to create a market niche and thus a basis for commercial investment in healthier options for managing fish production. For many skeptics, however, any organic standard that includes aquaculture is suspect.

George Leonard, director of the Aquaculture Program at the California field office of the nonprofit Ocean Conservancy, says the hurdle to enacting a sound organic standard involves resolving contradictions between the 1990 legislation creating the USDA organic label and environmentally sound seafood farming. These contradictions involve the difficulty adapting the legal concept of “organic” in the 1990 law, which was created for terrestrial agriculture, to the very different reality of aquaculture.

Leonard gives the example of farmed salmon, where he says the environmental impact of not using any antibiotics could in some cases endanger wild populations. “Disease is a primary area where there’s a mismatch between the principles of organic food and sustainability,” he explains. “From an organic consumer perspective, you’d usually want an outright ban on [antibiotics].” Yet that could put wild fish at greater risk of exposure to unhealthy farmed fish that may escape to the wild. He argues for a third path: the creation of a performance matrix for pen-fish farming that “sets a high environmental bar consistent with the Organic Food Act.”

## Fish in Human Health

“The fear of contaminants in fish is real, but not necessarily balanced with the actual risks,” says Jeff Silverstein, leader of the aquaculture program for the USDA Agricultural Research Service (ARS). “Certainly we now know that risk of contaminants is related to where the fish grow or are grown and what they eat.” In fact, farmed fish are usually smaller species such as tilapia, which have relatively short life spans in which to accumulate contaminants such as PCBs and other organochlorine compounds; these toxicants are more of a problem in longer-lived farmed fish.

But the use of anti-infective agents in farmed fish also poses a concern for critics of aquaculture. Urvashi Rangan, director of technical policy for the nonprofit Consumers Union, says, “Just in the past few years, there have been several documented accounts of farmed fish, . . . being sold to consumers around the world, that has been contaminated with banned antibiotics, carcinogenic fungicides, and coloring [agents].” For example, malachite green, a fungicide suspected to be a mutagen, teratogen, and carcinogen, has been banned internationally since the 1990s. Yet the compound has been found in some farmed (and wild) salmon.

In the 2005 report *Quantitative and Confirmatory Analyses of Malachite Green and Leucomalachite Green Residues in Fish and Shrimp*, scientists at the FDA reported finding malachite green in farm-raised tilapia, catfish, salmon, and trout. In a 20 November 2008 report to Congress on enhanced aquaculture and seafood inspection, the FDA documented that 7% of samples analyzed in 2007 contained restricted or banned substances including three antibiotics (chloramphenicol, nitrofurans, and fluoroquinolone) and two fungicides (malachite green and gentian violet). Most violations occurred in farmed fish imported from Asia—mainly China, Vietnam, and Indonesia—and South America.

Yet aquaculture’s supporters say critics exaggerate the use of malachite green and other anti-infectives. “It’s simply not true,” says George Lockwood, a member of the NOSB working group and an industry consultant who has seen aquaculture grow through nearly four decades. “There is very little antibiotic use today in any form of aquaculture.”

Silverstein says that, until recently, FDA had approved only Romet and oxytetracycline—two broad-spectrum antibiotics—for use, and that antibiotic use in salmon farming has “dropped tremendously with the use of vaccines.” Some sources, including a 2002 working paper from the Norwegian Institute for Research in Economics and Business Administration titled “Norwegian Salmon Aquaculture and Sustainability: The Relationship between Environmental Quality and Industry Growth,” maintain that proper siting of salmon farms and introduction of a vaccine against bacterial diseases make antibiotics “more or less redundant.”

Anti-infective use in aquaculture remains debatable, however, and some experts consider it an ongoing concern. Ned Daly, North American director for the Seafood Choices Alliance, a nonprofit coalition that involves aquaculture producers, environmental groups, and other stakeholders, admits that economic pressures for higher stocking densities push fish farmers toward antibiotics—for example to combat the bacteria carried by sea lice, parasites that feed primarily on salmon. Although Rust says some countries, such as Norway, do a creditable job of monitoring and disclosing antibiotic use, Daly maintains that “the amount of use is difficult to tell. . . . It’s one of the biggest issues in terms of human health.”

## Humans and Fish Health

Overexploitation of wild fisheries has been a problem for years (see “The State of the Oceans, Part 1: Eating Away at a Global Food Source,” *EHP* 112:A282–A291). Aquaculture has been cited as an option for reducing global reliance on the seas and reversing the decline of wild fish populations, but critics have long argued that aquaculture’s risks to ocean populations are more serious than the potential gains. In decades past, environmentalists expressed concern that some shrimp farms were being built in sensitive coastal wetlands and discharging excessive nutrients into estuaries. In a review in the 29 June 2000 issue of *Nature*, Rosamond L. Naylor and colleagues wrote that despite significant improvements, aquaculture represented a “threat, not only to ocean fisheries, but also to itself.”

Today’s shrimp farms are more tightly regulated, says George Chamberlain, president of the Global Aquaculture Alliance, a trade association, and many operate with little or no discharge of water. Aquaculturists contend that dependence on feeds made from wild fish–derived fishmeal is steadily declining with improved technology for use of vegetable proteins and rendered animal by-products fortified with crystalline amino acids—all while the production of aquaculture has almost doubled over the past decade, says Rust. In addition, alternative sources of long-chain fatty acids are also being sought out in the form of algal oils and genetically modified soybean oils.

But critics still express concern over the environmental impact of fish farms, particularly the system called “open-net pens,” practiced in open waters. Large nets essentially create a corral in which the fish grow, through which currents pass freely. Rangan calls these nets “concentration factories” for diseases such as sea lice, which can flourish in dense farm populations and spread to surrounding waters.

Sea lice infestations caused by salmon farms are cited as a potential limiting factor to wild fish conservation, according to a study by Martin Krkošek and colleagues published in the 7 April 2005 issue of *Proceedings of the Royal Society B*. That study’s model and a subsequent modification of it by the same authors (published online 6 May 2009 ahead of print in the same journal) indicated that an isolated salmon farm between two salmon migration routes in fjordic habitats of British Columbia, Canada, may raise the infection pressure for sea lice for 75 km along those routes. Although sea lice populations declined rapidly following brief exposure to juvenile salmon, longer-term exposure over several weeks as juvenile salmon migrate past salmon farms can elevate sea lice levels to the point that they can depress salmon populations.

Others contend that sea lice are naturally occurring parasites of marine fish whose abundance varies from year to year. In the November 2005 issue of *Fisheries Research*, R.J. Beamish and colleagues concluded that sea lice are a common parasite of adult Pacific salmon and are as likely to be found in the open sea as in a fish farm.

Rebecca Goldburg, a coauthor of the 2000 *Nature* review and now director of marine science for the Pew Environment Group, a nonprofit research arm of the Pew Charitable Trusts, sees aquaculture as a significant piece of the puzzle for addressing the threats to marine systems. “From my perspective, aquaculture is important for producing more food in the future,” she says. “That’s undeniable.” Some producers are eager for standards to validate practices they already use—she cites interest from salmon farmers, a few tilapia growers, and a farm off Hawaii raising amberjack. By setting a verifiable standard, she says policy-makers can create incentives for producers to innovate and substitute better fish feeds.

## Incentives for Innovation

Several federal agencies are helping to make that happen. ARS has collaborated with NOAA on alternatives to fish feed, fishmeal, and fish oils. Silverstein believes this work can yield such alternatives, ranging from plant proteins and insect meals to the large volume of fish by-products harvested every year. Useful by-products (heads, guts, and frames) from wild-harvest processing scraps from Alaska alone are estimated at up to 1 million metric tons a year, says Rust—enough to satisfy the fishmeal demand of the entire U.S. aquaculture industry. His office calculated that amount could feed roughly 1 billion farmed fish without using a single fish harvested solely for fishmeal.

The challenge is to prevent the by-product from rotting before it is processed into fishmeal, so NOAA research has focused on how to stabilize that material. Researchers add an acid to lower the pH and extend the by-product shelf life from a few days to three or four weeks. “Now [we’re] finding the minimum amount of acid we can get away with,” says Rust. He heads a team reporting on the USDA–NOAA collaboration on fish feeds; a draft for public comment is expected later this summer, with a final report near the end of the year.

The alternative to open systems is closed farms where there’s no potential for either nutrient effluent or fish to escape into surrounding waters. These involve fish tanks or ponds and systems for recirculating the water. In recent years the industry has explored recirculating systems, but they require higher energy and related costs. Growers have been slow to shift, especially for larger fish and the larger ponds they require. Looking ahead, Rust expects closed systems will become more efficient with innovative technologies.

He also predicts that open-water systems will evolve from near-shore fish farms to systems operated farther offshore, including submersible systems that Rust says are completely contained and very robust. “They’re shaped like a beach ball or a big top and can go underwater” to avoid storms and other disruptions, he says. These systems are still roughly twice as expensive as near-shore opennet pens, but just as the salmon farming industry led to innovations that made near-shore systems cost-effective, the same opportunity exists for offshore farms.

Some improvements in aquaculture involve genetics—even in reducing energy demand and chemical use. In mapping the genome of the rainbow trout (*Oncorhynchus mykiss*), for example, ARS researchers are identifying genes related to improved disease resistance, which can make for less reliance on anti-infectives. They may also find that a fish variety from a particular tributary or population experiences less stress in a closed-farm system, which may yield other benefits in the system, such as reduced requirements for energy, medications, or feed supplements.

## An Organic Standard

The global economic downturn has made progress in such areas uncertain in the near term, but if fish prices remain high, says Rust, that premium might fund innovation. Because the United States remains a small player in the global aquaculture sector, its greatest opportunity in organic fish production may lie in creating alternative fish feeds that resolve environmental concerns about fish feed sources and that engage the U.S. advantage in agricultural production for fish farms located overseas.

Daly predicts that an organic standard for aquaculture would have a ripple effect, influencing fish farmers when innovations for health yield greater efficiency, and making consumers more aware of their market choices. Since the United States imports around 80% of the fish it consumes, any U.S. standard will get the attention of suppliers abroad. “You could say organic is a niche market,” says Daly, “but it’s the fastest-growing market in the food sector, and that has a significant impact [on industrial practices overall].” Currently, organic goods constitute about 3% of the total agriculture market, and Lockwood predicts a similar bracket for organic aquaculture.

An organic standard for aquaculture would also bolster the role of the industry in meeting human health needs, says Lockwood. That includes providing sufficient amounts of the omega-3 fatty acids found mainly in fish oils. Lockwood says the NOSB working group made recommendations to the USDA covering all areas needed to ensure an organic standard: siting requirements; feed inspection; low stocking densities; certified sources of fish stock; production of meal and oil only from fish by-products; healthy facilities; and humane killing and transport procedures.

Under pressure from environmental groups, NOSB made last-minute changes to the criteria last November, requiring net-pen farms in open waters to capture and re-use 50% of all nitrogen and phosphorus metabolites produced in that system. That would cause problems for the industry, in Lockwood’s view—in systems where you can remove most of the phosphorus, it’s usually not feasible to re-use all nitrogen simultaneously. He suggests the criteria should instead mandate re-capture of the “limiting nutrient in the environment into which wastes are discharged for each system,” meaning the nutrient most scarce relative to needs in that environment—which in most marine systems is typically nitrogen.

Rangan believes the USDA process compromised by leaning too far toward industry. “The USDA has the opportunity to set high organic fish standards today, where certain fish—like shrimp and tilapia—could meet the current organic food standard and others—like salmon—could not. But that effort has been thwarted by those who want the bar to be set lower, arguing that increased market share justifies the proposed undermining of organic food standards and quality.”

Whereas an organic standard appears to be two years away from implementation by most estimates, other certification schemes should be coming to stores sooner. There are two leading contenders—one still in development by the nonprofit World Wildlife Fund and one started in 2003 by the Global Aquaculture Alliance. On the broader issue of healthy fish populations globally, Ocean Conservancy supports the World Wildlife Fund dialogues on sustainable aquaculture and a rigorous eco-certification standard, whereas certification of farms, hatcheries, and processing plants by the Global Aquaculture Alliance has momentum with support of Darden Restaurants (which owns Red Lobster, among other chains), Wal-Mart, and other retailers. The latter scheme is also being pilot tested by the FDA.

Either system should address the core elements of containment (prevention of escapes), feed, farm siting, stocking density, use of inputs such as anti-infectives, and chain-of-custody verification (meaning the process is inspected at each step from origin to consumer, with no gaps where contaminants or shoddy practices could intervene). Certifying chain of custody will be “a thousand times easier” for farmed fish than for wild fish, says Daly. Of the two schemes, Goldburg says, “Let the best one win—it doesn’t hurt to have a little competition.”

## Figures and Tables

**Figure:**
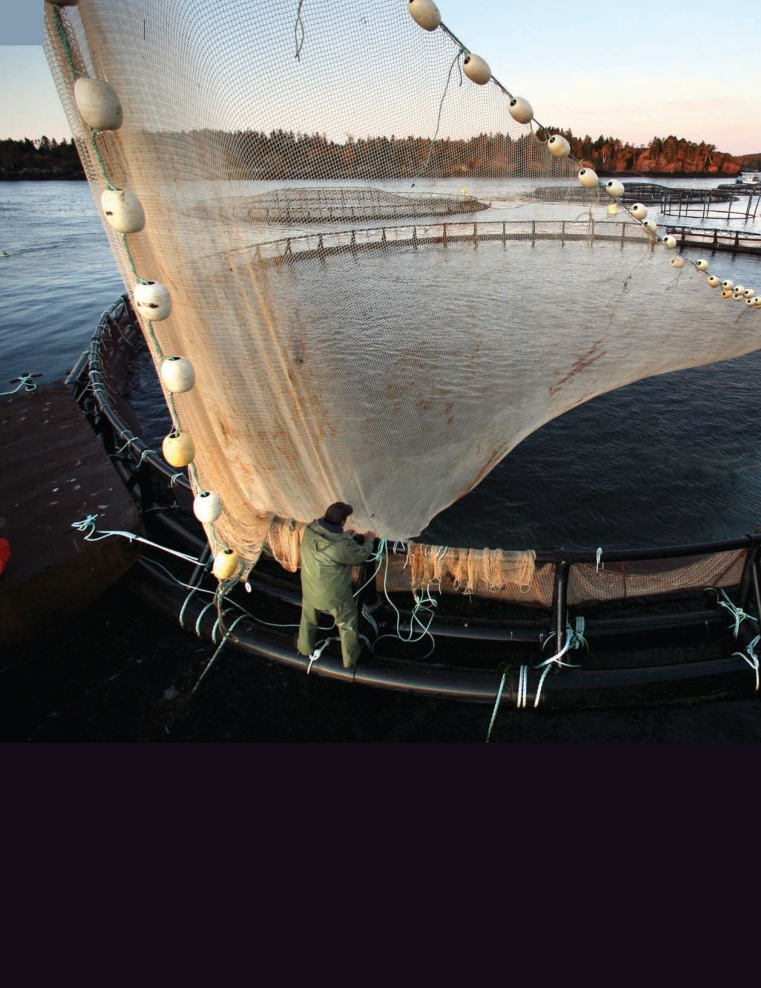
12 October 2008, a worker sets a net before harvesting salmon in a farm pen, Eastport, Maine. Many salmon farmers and other aquaculture operations would welcome standards that validate sustainable practices they already use. But putting such standards in place is easier said than done.

**Figure:**
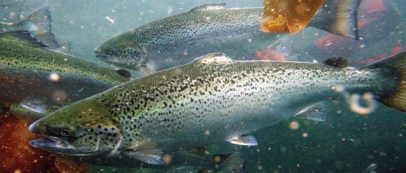
Atlantic salmon (*Salmo salar*)

